# Protocol for the development of a global core outcome set for the surgical treatment of sacrococcygeal teratoma in children: a systematic review and international Delphi study

**DOI:** 10.1136/bmjopen-2025-112492

**Published:** 2026-01-16

**Authors:** Malou Cécile Dongen, Rick van Rijn, Shilpa Sharma, Martine F Raphael, Ralph de Vries, Amr A. Abouzeid, Marianna Bugiani, Lohfa B Chirdan, Ernest L W van Heurn, Joep P M Derikx, Marijke E B Kremer, SCT-COS Steering Group

**Affiliations:** 1 Department of Paediatric Surgery, Emma Children’s Hospital, Amsterdam UMC, University of Amsterdam, Amsterdam, The Netherlands; 2 Amsterdam Reproduction & Development Research Institute, Amsterdam UMC, Amsterdam, The Netherlands; 3 Department of Radiology and Nuclear Medicine, Amsterdam UMC, Amsterdam, The Netherlands; 4 Department of Paediatric Surgery, All India Institute of Medical Sciences New Delhi, New Delhi, DL, India; 5 Department of Paediatric Oncology, Emma Children's Hospital, Amsterdam UMC, University of Amsterdam, Amsterdam, The Netherlands; 6 Medical Library, Vrije Universiteit, Amsterdam, The Netherlands; 7 Department of Paediatric Surgery, Ain Shams University, Cairo, Egypt; 8 Department of Pathology, Amsterdam UMC, Amsterdam, The Netherlands; 9 Department of Surgery, Paediatric Surgery Unit, Jos University Teaching Hospital, Jos, Plateau, Nigeria

**Keywords:** Paediatric surgery, Paediatric oncology, Delphi Technique, Systematic Review

## Abstract

**Abstract:**

**Introduction:**

Outcome reporting in studies on sacrococcygeal teratoma (SCT) is highly heterogeneous, which limits comparability across studies and thus hampers the development of international treatment guidelines.

Variation in treatment and access to facilities contributes to differences in outcome reporting between centres and countries. Establishing a Core Outcome Set (COS) can improve consistency in outcome reporting and facilitate global collaboration and data comparison. We therefore aim to develop a Core Outcome Set for SCT (COS-SCT) using the Delphi method to achieve consensus on key outcomes. This will enhance the standardisation of outcome reporting and improve the quality of research and clinical care for SCT patients globally.

**Methods and analysis:**

The development of the COS-SCT will consist of three phases. First, a systematic review will be performed to identify outcomes reported in studies on the surgical treatment of SCT in children. Second, an international Delphi survey will be conducted among key stakeholders, including clinicians, researchers and patient representatives, to establish consensus on outcome prioritisation. Finally, a consensus meeting with representatives from all stakeholder groups will be held to ratify the final Core Outcome Set. The study will follow methodological guidance from the Core Outcome Measures in Effectiveness Trials (COMET) initiative and will be developed and reported in accordance with the Core Outcome Set Standards for Development (COS-STAD) and Core Outcome Set Standards for Reporting (COS-STAR).

**Ethics and dissemination:**

The medical research ethics committee of the Amsterdam University Medical Centre (Amsterdam UMC) confirmed that the Dutch Medical Research Involving Human Subjects Act (WMO) does not apply to this study, and therefore a full review by the ethics committee is not required. This study is registered in the COMET initiative database. Results will be disseminated in peer-reviewed academic journals and conference presentations.

**Trial registration number:** COMET registration number 3485

**STRENGTHS AND LIMITATIONS OF THIS STUDY**

- This study follows established methodology for Core Outcome Set development.
- A broad range of stakeholder groups, including patient representatives and clinicians from different specialties, will contribute to the development of the Core Outcome Set.
- International participation is planned to support the applicability of the Core Outcome Set across different healthcare settings.
- A potential limitation is that stakeholder representation may vary across Delphi rounds.

## Introduction

 Sacrococcygeal teratoma (SCT) is a rare congenital neoplasm, occurring in approximately 1 in 14 000–40 000 live births worldwide.^1 2^ Although the majority of SCTs are benign at birth, malignant transformation may occur, particularly in cases diagnosed postnatally or when resection is delayed or incomplete.^3^ Complete surgical resection, including removal of the coccyx, is considered standard of care and essential for adequate tumour control.^4^ Patients diagnosed postnatally generally have a favourable prognosis following early surgical resection*,* with reported survival rates between 89% and 95%. ^5 6^ However, long-term outcomes are variable due to the risk of functional sequelae after treatment.^7 8^ A major challenge in SCT research is the inconsistency in outcome reporting. Studies differ in which outcomes are selected and how they are defined, and there is no consensus on which outcomes are most critical for evaluating treatment and follow-up. This heterogeneity limits comparability across studies and impedes data synthesis.^9^ The rarity of SCT further complicates this issue, as most studies involve small study cohorts and prolonged inclusion periods, reducing their statistical power and generalisability. As a result, researchers often need to combine data across international cohorts to draw meaningful conclusions. A Core Outcome Set (COS) is a standardised group of outcomes that should be measured and reported in all studies on a specific condition.^10^ A COS provides a framework for consistent outcome selection and reporting. This enhances comparability and improves the quality of research.^11^ Currently, no COS exists for the surgical treatment of SCT. We aim to reach a global consensus among clinicians, researchers and patient representatives on the minimum set of outcomes that should be measured and reported in future research on surgical treatment for SCT in children.

### Methods and analysis

### Study design

**Figure 1:**
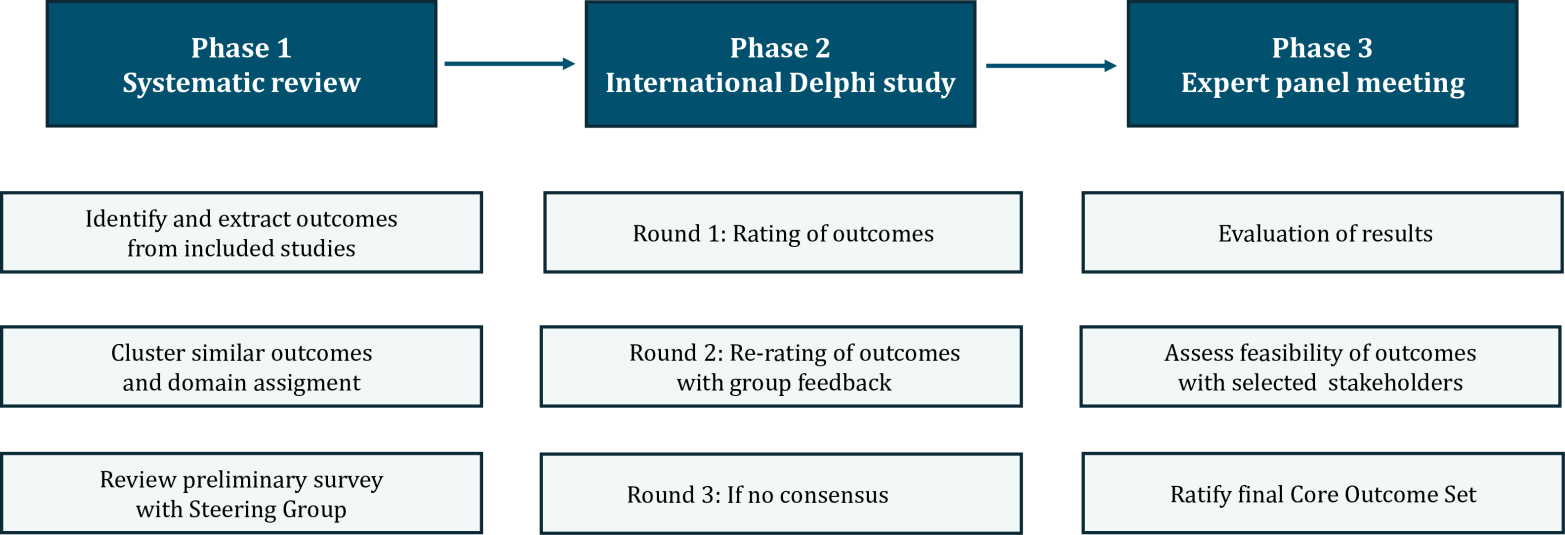
Overview of the study design

The sacrococcygeal teratoma Core Outcome Set (SCT-COS) will be developed in three consecutive phases. First, a systematic review will be conducted to identify outcomes reported in studies on the surgical treatment of SCT for children. Second, an international Delphi study will be conducted to prioritise the outcomes identified in the review. During the last phase, a consensus expert meeting will be held to ratify the final COS and facilitate its implementation in clinical research. The COS is intended to be globally applicable and feasible for use in both high and low resource healthcare settings. This will be supported by broad international stakeholder recruitment. All stakeholder groups will participate in the same Delphi process. Where relevant, stratified analysis will be conducted to explore variation in outcome prioritisation across stakeholder groups and regions. The development and reporting of this protocol will follow the COS-STAD (Core Outcome SET Standards for Development) recommendations and the COMET (Core Outcome Measures in Effectiveness for Trials) initiative.^12^ This study has been registered in the COMET database (registration number 3825, registered on 2 July 2025). The definitive core outcome set will be reported according to the Core Outcome Set-Standards (COS-STAR) for reporting statement.^10^ This protocol follows the SPIRIT 2013 Statement for clinical study protocols, and the completed checklist is available in online supplemental file 1. The Delphi study is planned to commence in early 2026. An overview of the study design is shown in figure 1.

### Study steering group and management group

The study management group will consist of Prof. L.W.E. van Heurn (paediatric surgeon), Prof. J.PM. Derikx (paediatric surgeon), Dr. M.E.B. Kremer (paediatric surgeon) and M.C. Dongen (MD, PhD candidate). The group will be responsible for the overall coordination of the project, including protocol development, conduct of the systematic review, management of the Delphi process and organisation of the final consensus meeting. Regular meetings will be held throughout the project to discuss the progress. The study steering group will consist of professionals from a range of regions and specialties involved in the care of children with sacrococcygeal teratoma. The steering group will agree on the final version of the protocol at the start of the project and will oversee the Delphi process in varied healthcare contexts. Members will be involved in participant selection and provide feedback during the study. To avoid bias, steering group members will not complete the Delphi survey. They will participate in the final consensus meeting to help ensure that the Core Outcome Set is relevant, feasible and reflects the perspectives of all key stakeholders.

### Sample size

There is no universally accepted sample size for panel members in a Delphi study.^9 13^ However, a minimum of seven participants per stakeholder group is generally recommended to ensure sufficient diversity and enable consensus, particularly given the risk of attrition between rounds.^14^ We aim to invite approximately 20–30 participants per stakeholder group (eg, paediatric surgeons, paediatricians, patient representatives, radiologists and pathologists), with representation from both high-resource and low-resource settings. This is expected to result in a total sample size of around 100 to 150 participants, allowing for a 20% rate of non-responders or loss to attrition. In case the number of respondents per country is significantly higher than others, we will consider a weighting by country in the analyses. There will be no maximum number of respondents.

### Data management

All data will be handled confidentially and in accordance with the European General Data Protection Regulation and the Dutch Act on Implementation of the General Data Protection Regulation. The Delphi study will be conducted online and managed using Welphi software.

### Phase 1

#### Systematic review

##### Search strategy

A comprehensive literature search will be performed in Ovid Medline, Embase.com and Web of Science (Core Collection). The search strategy was developed in collaboration with a medical librarian and included controlled vocabulary (eg, MeSH) and free-text terms related to “sacrococcygeal teratoma”, “surgery” and “outcomes”. Specific attention will be given to the inclusion of studies from low-resource settings. The systematic review will be conducted in accordance with the Preferred Reporting Items for Systematic Reviews and Meta-Analyses (PRISMA) guideline, and the protocol has been registered in PROSPERO (registration number: CRD42025618725). The full search strategy is available in online supplemental file 2.

### Inclusion and exclusion criteria

Retrospective and prospective cohort studies and case series reporting on surgical outcomes in patients aged 0–18 years with sacrococcygeal teratoma will be included in this review. Studies will be excluded if they focus on foetal interventions, report only foetal outcomes, do not clearly describe surgical treatment, or report only on recurrent SCT. Foetal interventions will be excluded as they are not representative of postnatal surgical management. Studies published before 1980 will be excluded, as Currarino syndrome had not yet been recognised as a separate diagnosis. This condition, which includes anorectal malformations and sacral anomalies, involves a distinct clinical course and a lower risk of malignancy and is therefore not comparable to isolated SCT.^15^ Single case reports, studies with fewer than five patients, non-English publications and studies published in abstract form only will also be excluded.

### Data extraction

Data will be systematically extracted from the included studies using a structured spreadsheet. Two authors (MD, MK) will independently perform the screening and selection of articles, beginning with title and abstract review (Level 1) and progressing to full-text screening (Level 2). Extracted data will include study characteristics (author, year, study design, sample size and study period) and all reported outcomes. When provided, definitions used for each outcome will also be collected. A third reviewer (JD) will be consulted in case of disagreement. Only outcomes explicitly reported in the results sections of the included studies will be considered for inclusion.

### Data synthesis

All outcomes will be categorised into the following core areas: Life impact, death, pathophysiological manifestations, resource use and adverse events following the OMERACT filter 2.0 method.^16^ Table 1 presents the classification of outcomes according to the OMERACT filter 2.0. To avoid duplication and facilitate comparison, conceptually similar terms describing the same intended construct will be grouped into unified outcomes. For example, the outcomes ‘soiling’, ‘encopresis’ and ‘faecal incontinence’ will be grouped together under the unified term ‘faecal incontinence’. The clustering of outcomes will be performed independently by two reviewers (MD and JD), with discussion until consensus is reached. Adverse events will be labelled separately within their core area. The preliminary list of unified outcomes will be presented to the steering group for review and subsequently used to construct the Delphi questionnaire.

**Table 1:** Classification of outcomes according to the OMERACT filter 2.0 method

Core area	Examples
Death	Disease-specific survival
Life impact	Quality of life, patient perception of health
Resource use	Healthcare costs, length of hospital stay
Pathophysiological manifestations	Oncological manifestations, eg, recurrence
Adverse events	Surgical complications

### Participants: panel selection

To develop a globally relevant Core Outcome Set for the surgical treatment of SCT, stakeholders from both high-resource and low-resource settings will be included. Antenatal diagnostic practices differ substantially between settings. These differences may influence clinical presentation and the feasibility or timing of treatment, underlining the importance of broad international representation. To ensure this, we will involve a broad range of healthcare professionals in the development of the COS.

### Patient representatives

To ensure that the perspectives of patients and their families are reflected in the COS for SCT, we will actively involve both children and parents. Literature indicates that children and caregivers are not consistently included in COS development, despite potentially differing priorities from healthcare professionals.^17^ Children and adolescents aged 12 to 18 years who underwent surgical resection for SCT during the neonatal period will be included. Children younger than 12 years will be excluded due to the complexity of the methodology and survey design. Adolescents will be encouraged to complete the questionnaire with their caregivers. Recruitment of children and parents will be coordinated through the databases of the Emma Children’s Hospital and the Follow Me outpatient clinic. International recruitment will occur through the European reference network for paediatric oncology and surgery, as well as through patient advocacy groups where available.

### Healthcare professionals

#### Paediatric surgeons

Paediatric surgeons involved in the care of patients with SCT will be included as the largest stakeholder group due to their central role in surgical management and long term follow-up. Participants will be recruited through established professional networks, such as the European Paediatric Surgeons’ Association (EUPSA) and affiliated national organisations, as well as through relevant conferences.

#### Paediatric oncologists, neonatologists and other paediatricians

Paediatric oncologists, neonatologists and paediatricians will be included as stakeholder groups, reflecting their respective roles across the clinical course of SCT. Paediatric oncologists are involved in the management of malignant disease. Neonatologists provide care in the perioperative and early postoperative period. During long-term follow-up, some children may be seen by paediatricians for bowel function or general developmental concerns, as they frequently serve as the first point of contact outside the tertiary setting. Recruitment will occur through professional networks, institutional contacts and relevant societies such as the International Society of Paediatric Oncology (SIOP).

#### Paediatric urologists

Paediatric urologists will be included as a stakeholder group, as urological sequelae after SCT resection are frequently reported and are relevant in long-term follow-up. Participants will be recruited through professional networks, institutional contacts and relevant paediatric urology associations.

#### Pathologists and radiologists

Radiologists and pathologists will be included as separate stakeholder groups due to their contribution to diagnostic evaluation and monitoring recurrence during follow-up. Participants will be approached through networks such as the European Society of Paediatric Radiology (ESPR), as well as national and institutional contacts.

### Phase 2: International online Delphi Study

The Delphi method is an established approach for achieving consensus within a large group without requiring face-to-face interaction.^13^ Participants from all stakeholder groups will complete iterative rounds consisting of anonymous questionnaires administered via Welphi Manager, a specialised web-based platform for conducting Delphi studies. The questionnaire will be initially formulated into English and will be translated into other languages if necessary. The list of outcomes obtained from the systematic review will be formatted into questions for the Delphi questionnaire. Participants will be asked to score the importance of each outcome for inclusion in the Core Outcome Set, using a 1-point to 9-point Likert scale, recommended by the Grading of Recommendations Assessment, Development and Evaluation (GRADE) working group and the COMET initiative.^9 18^ A score of 7–9 indicates that an outcome is considered critically important regarding treatment and should be included in the COS. A score of 4–6 indicates that the outcome is considered important but not critical and 1–3 indicates the outcome is not important for evaluating the effect on treatment and should not be included in the COS. Additionally, there will be the option to answer ‘unable to score’ or ‘not my expertise’ for participants who feel they lack the knowledge or experience to accurately assess certain outcomes. We will develop an equivalent questionnaire with lay terms for the patient representative stakeholder group. Before the first Delphi round, the lay version of the questionnaire will be piloted with a small group of parents to ensure the items are clear and relevant to their experiences with SCT. Parents will be invited to comment on any aspects they find unclear or where important outcomes may be missing. Their feedback will help refine the questionnaire before the first Delphi round. As part of the testing phase, both questionnaires will be reviewed to assess the readability and identify any ambiguous questions. Prior to the first Delphi round, all the invited participants in this study will be provided with information on the study. Participants have the option to disengage from the study at any time throughout its duration. If participants do not complete a round in the Delphi study, they are not eligible to proceed to the next voting round or to participate in the final consensus meeting.

The Delphi survey will be conducted anonymously. Participants will not see individual scores from other panel members at any point. All scoring will be completed independently by each participant.

### Delphi round 1

All participants will be asked to provide basic demographics (age, gender and country). Medical professionals will be asked to specify their specialism and their workplace (academic or referral centre for SCT, teaching hospital or non-teaching hospital) and whether they are involved in SCT research. The patient stakeholder group will be asked to provide their educational level, time passed since their treatment and overall experience during their treatment. Participants will be asked to rate the importance of each outcome for inclusion in the Core Outcome Set. The Delphi survey will ask participants the following question: ‘*How important is this outcome for inclusion in a Core Outcome Set?’* Each outcome will be rated on a 9-point scale: 1 to 3 (not important), 4 to 6 (important, but not critical) and 7 to 9 (critical). As previously mentioned, all outcomes will be categorised into one of the five pre-determined core areas. Following the completion of the first round, participants will have the opportunity to propose additional outcomes they believe are important for the Core Outcome Set, if they are not included in the first round. A period of 3 weeks will be scheduled for the completion of each round. If stakeholders have not returned their completed questionnaire within 2 weeks, they will receive a reminder by email.

### Delphi round 1: Analysis

The results of the first Delphi round will be analysed separately for participants from both high-income and low-income settings. Additionally, all results will be evaluated for each stakeholder group using descriptive statistics.

‘Consensus-in’ will be defined as follows:

- Greater than 70% of participants from both stakeholder groups (excluding those who select ‘Not my area of expertise’) rate the outcome as 7–9, with fewer than 15% rating it as 1–3.
- Greater than 90% of participants within a single stakeholder group rate the outcome as 7–9. This ensures that outcomes important to only one stakeholder group can still be included.

Greater than 70% of participants from both stakeholder groups (excluding those who select ‘Not my area of expertise’) rate the outcome as 7–9, with fewer than 15% rating it as 1–3.

Greater than 90% of participants within a single stakeholder group rate the outcome as 7–9. This ensures that outcomes important to only one stakeholder group can still be included.

‘Consensus out’ will be defined as follows:

- Greater than 70% of participants in both stakeholder groups rate the outcome as 1–3, and less than 15% participants in both stakeholder groups rate the outcome as 7–9. Consensus can only be reached if there is consensus across both stakeholder groups.

Following the analysis of the first round, the study management group will assess whether any new outcomes suggested by respondents should be incorporated as additional outcomes. Questions will be rephrased if there is evidence of potential misinterpretation. A stratified analysis may be conducted to identify and address potential skewing caused by divergent opinions from specific subgroups. If such skewing is detected, corrective adjustments will be applied to the analysis.

### Delphi round 2 and 3

All participants who have completed the first round will be invited to participate in the second Delphi round. Only outcomes that have not yet been defined as ‘consensus in’ or ‘consensus out’ will be presented to all participants in the second round. Any newly suggested outcomes from the previous rounds will also be included for all participants. Outcomes for which consensus is achieved within a single stakeholder group will be presented to the other stakeholder groups to determine whether consensus can be achieved among all stakeholder groups. In round two, participants will be presented with a histogram displaying the distribution of scores and the median score from the responses of other participants, alongside a reminder of their individual response from round one. Participants will then be asked to re-evaluate and rate the outcomes in the same manner as in the first round.

### Delphi round 2 and 3: analysis

The results will be analysed separately for each stakeholder group as well as for all participants, using descriptive statistics and stratified analysis. The same criteria for consensus inclusion and exclusion as defined in the first Delphi round will be applied. Following the second round, the study management and steering group will evaluate whether modifications to the Delphi process are necessary and decide on the need for a third Delphi round. This decision will be based on achieving consensus between both stakeholder groups on more than 80% of the outcomes and having at least five outcomes reach consensus inclusion.

### Phase 3: Development of the final COS

#### Expert panel meeting

Once consensus is reached through the Delphi process, an expert panel meeting will be organised to ratify a pragmatic and well-defined set of outcomes and to promote its implementation. A formal in-person consensus meeting will not be organised to avoid selection bias among participants who are able to attend. If consensus cannot be reached in the Delphi process on at least one outcome per core domain, a virtual meeting with broad stakeholder representation will be held. The panel will consist of selected representatives across stakeholder groups, with approximately 15–20 participants from the ‘professionals’ stakeholder group identified through purposive sampling. This will not involve all Delphi participants, but a smaller panel aimed at assessing the feasibility of the Delphi results in clinical practice. Patient representatives and parents may also be invited to contribute remotely. The meeting will either take place during an international paediatric surgery conference or be conducted via videoconference to accommodate broader participation.

Our Delphi study aims to develop a pragmatic COS that is feasible for use in future clinical research and in evaluating outcomes of SCT treatment. In this final phase, only outcomes meeting the following criteria will be included:

1. Relevant: The outcome must address a key aspect of SCT care and be deemed important by stakeholders.
2. Measurability: The outcome must be assessable using established and validated tools or instruments.
3. Specificity: The outcome must be directly modifiable by interventions targeting SCT.

Relevant: The outcome must address a key aspect of SCT care and be deemed important by stakeholders.

Measurability: The outcome must be assessable using established and validated tools or instruments.

Specificity: The outcome must be directly modifiable by interventions targeting SCT.

The final COS will include a minimum of one outcome per OMERACT core domain and will consist of between 8 and 15 outcomes. If consensus is achieved for more than 15 outcomes, the 15 outcomes with the highest levels of consensus will be prioritised for inclusion. The level of consensus for each outcome will be determined by agreement between both stakeholder groups, the median score assigned to the outcome and the IQR as a measure of the extent of agreement.

### Strengths and limitations of this study

There is no uniformity in evaluating treatment success and follow-up for patients with sacrococcygeal teratoma. Research in SCT is limited by studies with small sample sizes, long inclusion periods, heterogeneity in outcome reporting and differences across various healthcare settings, which makes it hard to synthesise data and make conclusions. Development of a Core Outcome Set will help standardise outcome reporting in future research and thereby improve data synthesis and overall quality.

### Ethics and dissemination

The medical research ethics committee of the Amsterdam University Medical Centre confirmed that the Dutch Medical Research Involving Human Subjects Act (WMO) does not apply to this study and thereby full review by the ethics committee is not required. Electronic informed consent will be obtained from all participants.

## Supplementary material

10.1136/bmjopen-2025-112492 online supplemental file 1

10.1136/bmjopen-2025-112492 online supplemental file 2

## Data Availability

No data are available.
