# Supplementary material for: Protocol for the development of a global core outcome set for the surgical treatment of sacrococcygeal teratoma in children: a systematic review and international Delphi study
Source: BMJ Open. 2026 Jan 16;16(1):e112492. doi: 10.1136/bmjopen-2025-112492 (PMC12815119; doi:10.1136/bmjopen-2025-112492)
Supplement: online supplemental file 2 [file bmjopen-16-1-s002.pdf]

Supplementary material 1. Search strategy

Ovid Medline Search Results - 19 Jun 2025

Search	Query	Results
#9	7 and 8	30
#8	"Developing Countries"/ or ("developing countr*" or "non-western" or "nonwestern" or "emerging econom*" or "developing nation*" or "developing population*" or "developing econom*" or "undeveloped countr*" or "undeveloped nation*" or "undeveloped economy" or "undeveloped economies" or "least developed countr*" or "least developed nation*" or "least developed economy" or "least developed economies" or "less-developed countr*" or "less-developed nation*" or "less-developed population" or "less-developed populations" or "less-developed econom*" or "lesser developed countr*" or "lesser developed nation*" or "lesser developed population" or "lesser developed populations" or "lesser developed economy" or "lesser developed economies" or "under-developed countr*" or "under-developed nation*" or "underdeveloped countr*" or "underdeveloped nation*" or "underdeveloped population*" or "underdeveloped econom*" or "low income countr*" or "middle income countr*" or "low income nation*" or "middle income nation*" or "low income population*" or "middle income population*" or "low income econom*" or "middle income econom*" or "lower income countr*" or "lower income nation*" or "lower income population*" or "lower income economy" or "lower income economies" or "resource limited" or "low resource countr*" or "lower resource countr*" or "low resource nation*" or "low resource population*" or "low resource economy" or "low resource economies" or "underserved countr*" or "underserved nation*" or "underserved population*" or "underserved economy" or "underserved economies" or "under-served country" or "under-served countries" or "under-served nation" or "under-served nations" or "under-served population" or "under-served populations" or "underserved economy" or "underserved economies" or "derived countr*" or "deprived nation" or "deprived nations" or "derived population*" or "deprived economy" or "deprived economies" or "poor countr*" or "poor nation*" or "poor population*" or "poor econom*" or "poorer countr*" or "poorer nation*" or "poorer population*" or "poorer econom*" or "lmic" or "lmics" or "lami" or "transitional countr*" or "transitional nation" or "transitional nations" or "transitional econom*" or "transition countr*" or "transition nation*" or "transition econom*" or "low resource setting*" or "lower resource setting*" or "middle resource setting*" or "Third World*" or Afghan* or Alban* or Algeria* or Angol* or Argentin* or Armenia* or Azerbaijan* or Bangladesh* or Belarus* or Belize* or Benin or Bhutan* or Bolivia* or Bosnia* or Botswan* or Brazil* or "Burkina Faso" or Burundi* or "Cabo Verd*" or Cambodia* or Cameroon* or "Central African Republic" or Chad or China or Colombia* or Comoros or Congo* or "Costa Rica*" or "Côte d’Ivoire" or Cuba* or	1,934,072

Search	Query	Results
	"Republic of Korea" or Djibouti* or Dominica* or "East Timor" or Ecuador* or Egypt* or "El Salvador*" or Eritrea* or Eswatini* or Ethiopia* or Fiji or Gabon* or Gambia* or "Gaza Strip" or (Georgia NOT state) or Ghan* or Grenada or Guatemal* or (Guinea NOT ("guinea pig*")) or Guyan* or Haiti* or Herzegovin* or Hondur* or India* or Indones* or Iran* or Iraq* or "Ivory Coast" or Jamaica* or Jordan* or Kazakhstan* or Kenya* or Kiribat* or Kosov* or Kyrgyzstan* or Lao* or Leban* or Lesoth* or Liberia* or Libya* or Madagascar* or Malaw* or Malaysi* or Maldives or Mali or Malian or "Marshall Islands" or Mauritani* or Mauriti* or Mexic* or Micronesi* or Mocambiqu* or Moldov* or Mongoli* or Monteneg* or Montserrat or Morocc* or Mozambiqu* or Myanmar or Namibi* or Nauru or Nepal* or Nicaragu* or Niger or Nigeria* or Niue or "North Korea*" or "North Macedoni*" or Pakistan* or Palau* or Panam* or Paragua* or Peru* or Philippin* or Rhodesia* or Rwand* or "Saint Helena" or "Saint Lucia" or "Saint Vincent" or "St Helena" or "St Lucia" or "St Vincent" or Samoa* or "Sao Tome" or Senegal* or Serbia* or "Sierra Leon*" or "Solomon Islands" or Somali* or "South Africa*" or "Southern Africa*" or "Sri Lank*" or "Sub Saharan Africa*" or "Subsaharan Africa*" or Sudan* or Surinam* or Syria* or Tajikistan or Tanzani* or Thai* or "Timor-Leste" or Togo* or Tokelau or Tonga or Tunis* or Turki* or Turkmenistan or Tuvalu or Ugand* or Ukrain* or Uzbekistan or Vanuatu or Venezuel* or "Viet Nam*" or Vietnam* or "Wallis and Futuna" or "West Bank" or Yemen* or Zambia* or Zimbabw*).ab,ti,kf.	
#7	6 not ("prenatal*" or "pre-natal*" or "fetal" or "foetal" or "fetus*" or "foetus*").ti.	904
#6	4 and 5	1,111
#5	("resect*" or "treat*" or "surg*" or "operat*" or "invasive" or "therap*" or "chemo*" or "manag*").ab,ti,kf.	12,891,426
#4	(1 and 2) or 3	1,950
#3	(("sacrococcygeal" or "coccygeal" or "sacral") adj3 "germ cell*").ab,ti,kf.	37
#2	"Sacrococcygeal Region"/ or ("sacrococcygeal" or "coccygeal" or "sacral").ab,ti,kf.	27,487
#1	exp "Teratoma"/ or "Teratocarcinoma"/ or "terato*".ab,ti,kf.	55,739

Search	Query	Results
#11	#9 NOT ('conference abstract'/it OR 'conference review'/it) NOT 'clinical trial':dtype	43
#10	#7 NOT ('conference abstract'/it OR 'conference review'/it) NOT 'clinical trial':dtype	1,007
#9	#7 AND #8	75
#8	'developing country'/exp OR 'low income country'/exp OR 'middle income country'/exp OR ("developing countr*" OR "non-western" OR "nonwestern" OR "emerging econom*" OR "developing nation*" OR "developing population*" OR "developing econom*" OR "undeveloped countr*" OR "undeveloped nation*" OR "undeveloped economy" OR "undeveloped economies" OR "least developed countr*" OR "least developed nation*" OR "least developed economy" OR "least developed economies" OR "less-developed countr*" OR "less-developed nation*" OR "less-developed population" OR "less-developed populations" OR "less-developed econom*" OR "lesser developed countr*" OR "lesser developed nation*" OR "lesser developed population" OR "lesser developed populations" OR "lesser developed economy" OR "lesser developed economies" OR "under-developed countr*" OR "under-developed nation*" OR "underdeveloped countr*" OR "underdeveloped nation*" OR "underdeveloped population*" OR "underdeveloped econom*" OR "low income countr*" OR "middle income countr*" OR "low income nation*" OR "middle income nation*" OR "low income population*" OR "middle income population*" OR "low income econom*" OR "middle income econom*" OR "lower income countr*" OR "lower income nation*" OR "lower income population*" OR "lower income economy" OR "lower income economies" OR "resource limited" OR "low resource countr*" OR "lower resource countr*" OR "low resource nation*" OR "low resource population*" OR "low resource economy" OR "low resource economies" OR "underserved countr*" OR "underserved nation*" OR "underserved population*" OR "underserved economy" OR "underserved economies" OR "under-served country" OR "under-served countries" OR "under-served nation" OR "under-served nations" OR "under-served population" OR "under-served populations" OR "underserved economy" OR "underserved economies" OR "derived countr*" OR "deprived nation" OR "deprived nations" OR "derived population*" OR "deprived economy" OR "deprived economies" OR "poor countr*" OR "poor nation*" OR "poor population*" OR "poor econom*" OR "poorer countr*" OR "poorer nation*" OR "poorer population*" OR "poorer econom*" OR "lmic" OR "lmics" OR "lami" OR "transitional countr*" OR "transitional nation" OR "transitional nations" OR "transitional econom*" OR "transition countr*" OR "transition nation*" OR "transition econom*" OR "low resource setting*" OR "lower resource setting*" OR "middle resource setting*" OR "Third World*" OR "Afghan*" OR "Alban*" OR "Algeria*" OR "Angol*" OR "Argentin*" OR "Armenia*" OR	2,477,222

Search	Query	Results
	"Azerbaijan*" OR "Bangladesh*" OR "Belarus*" OR "Belize*" OR "Benin" OR "Bhutan*" OR "Bolivia*" OR "Bosnia*" OR "Botswan*" OR "Brazil*" OR "Burkina Faso" OR "Burundi*" OR "Cabo Verd*" OR "Cambodia*" OR "Cameroon*" OR "Central African Republic" OR "Chad" OR "China" OR "Colombia*" OR "Comoros" OR "Congo*" OR "Costa Rica*" OR "Côte d Ivoire" OR "Cuba*" OR "Republic of Korea" OR "Djibouti*" OR "Dominica*" OR "East Timor" OR "Ecuador*" OR "Egypt*" OR "El Salvador*" OR "Eritrea*" OR "Eswatini*" OR "Ethiopia*" OR "Fiji" OR "Gabon*" OR "Gambia*" OR "Gaza Strip" OR ("Georgia" NOT "state") OR "Ghan*" OR "Grenada" OR "Guatemal*" OR ("Guinea" NOT ("guinea pig*")) OR "Guyan*" OR "Haiti*" OR "Herzegovin*" OR "Hondur*" OR "India*" OR "Indones*" OR "Iran*" OR "Iraq*" OR "Ivory Coast" OR "Jamaica*" OR "Jordan*" OR "Kazakhstan*" OR "Kenya*" OR "Kiribat*" OR "Kosov*" OR "Kyrgyzstan*" OR "Lao*" OR "Leban*" OR "Lesoth*" OR "Liberia*" OR "Libya*" OR "Madagascar*" OR "Malaw*" OR "Malaysi*" OR "Maldives" OR "Mali" OR "Malian" OR "Marshall Islands" OR "Mauritani*" OR "Mauriti*" OR "Mexic*" OR "Micronesi*" OR "Mocambiqu*" OR "Moldov*" OR "Mongoli*" OR "Monteneg*" OR "Montserrat" OR "Morocc*" OR "Mozambiqu*" OR "Myanmar" OR "Namibi*" OR "Nauru" OR "Nepal*" OR "Nicaragu*" OR "Niger" OR "Nigeria*" OR "Niue" OR "North Korea*" OR "North Macedoni*" OR "Pakistan*" OR "Palau*" OR "Panam*" OR "Paragua*" OR "Peru*" OR "Philippin*" OR "Rhodesia*" OR "Rwand*" OR "Saint Helena" OR "Saint Lucia" OR "Saint Vincent" OR "St Helena" OR "St Lucia" OR "St Vincent" OR "Samoa*" OR "Sao Tome" OR "Senegal*" OR "Serbia*" OR "Sierra Leon*" OR "Solomon Islands" OR "Somali*" OR "South Africa*" OR "Southern Africa*" OR "Sri Lank*" OR "Sub Saharan Africa*" OR "Subsaharan Africa*" OR "Sudan*" OR "Surinam*" OR "Syria*" OR "Tajikistan" OR "Tanzani*" OR "Thai*" OR "Timor-Leste" OR "Togo*" OR "Tokelau" OR "Tonga" OR "Tunis*" OR "Turki*" OR "Turkmenistan" OR "Tuvalu" OR "Ugand*" OR "Ukrain*" OR "Uzbekistan" OR "Vanuatu" OR "Venezuel*" OR "Viet Nam*" OR "Vietnam*" OR "Wallis and Futuna" OR "West Bank" OR "Yemen*" OR "Zambia*" OR "Zimbabw*"):ab,ti,kw	
#7	#6 NOT ("prenatal*" OR "pre-natal*" OR "fetal" OR "foetal" OR "fetus*" OR "foetus*"):ti	1,269
#6	#4 AND #5	1,533
#5	("resect*" OR "treat*" OR "surg*" OR "operat*" OR "invasive" OR "therap*" OR "chemo*" OR "manag*"):ab,ti,kw	17,794,086
#4	(#1 AND #2) OR #3	2,435
#3	(("sacrococcygeal" OR "coccygeal" OR "sacral") NEAR/3 "germ cell*"):ab,ti,kw	54

Search	Query	Results
#2	'sacrococcygeal region'/exp OR ("sacrococcygeal" OR "coccygeal" OR "sacral"):ab,ti,kw	38,781
#1	'teratoma'/exp OR 'teratocarcinoma'/exp OR "terato*":ab,ti,kw	82,331

Web of Science (Core Collection) Search Results – 19 Jun 2025

Search	Query	Results
#9	#7 AND #8	37
#8	TS=("developing countr*" OR "non-western" OR "nonwestern" OR "emerging econom*" OR "developing nation*" OR "developing population*" OR "developing econom*" OR "undeveloped countr*" OR "undeveloped nation*" OR "undeveloped economy" OR "undeveloped economies" OR "least developed countr*" OR "least developed nation*" OR "least developed economy" OR "least developed economies" OR "less-developed countr*" OR "less-developed nation*" OR "less-developed population" OR "less-developed populations" OR "less-developed econom*" OR "lesser developed countr*" OR "lesser developed nation*" OR "lesser developed population" OR "lesser developed populations" OR "lesser developed economy" OR "lesser developed economies" OR "under-developed countr*" OR "under-developed nation*" OR "underdeveloped countr*" OR "underdeveloped nation*" OR "underdeveloped population*" OR "underdeveloped econom*" OR "low income countr*" OR "middle income countr*" OR "low income nation*" OR "middle income nation*" OR "low income population*" OR "middle income population*" OR "low income econom*" OR "middle income econom*" OR "lower income countr*" OR "lower income nation*" OR "lower income population*" OR "lower income economy" OR "lower income economies" OR "resource limited" OR "low resource countr*" OR "lower resource countr*" OR "low resource nation*" OR "low resource population*" OR "low resource economy" OR "low resource economies" OR "underserved countr*" OR "underserved nation*" OR "underserved population*" OR "underserved economy" OR "underserved economies" OR "under-served country" OR "under-served countries" OR "under-served nation" OR "under-served nations" OR "under-served population" OR "under-served populations" OR "underserved economy" OR "underserved economies" OR "derived countr*" OR "deprived nation" OR "deprived nations" OR "derived population*" OR "deprived economy" OR "deprived economies" OR "poor countr*" OR "poor nation*" OR "poor population*" OR "poor econom*" OR "poorer countr*" OR "poorer nation*" OR "poorer population*" OR "poorer econom*" OR "lmic" OR "lmics" OR "lami" OR "transitional countr*" OR "transitional nation" OR "transitional nations" OR "transitional econom*" OR "transition countr*" OR "transition nation*" OR "transition econom*" OR "low resource setting*" OR	5,114,387

Search	Query	Results
	"lower resource setting*" OR "middle resource setting*" OR "Third World*" OR "Afghan*" OR "Alban*" OR "Algeria*" OR "Angol*" OR "Argentin*" OR "Armenia*" OR "Azerbaijan*" OR "Bangladesh*" OR "Belarus*" OR "Belize*" OR "Benin" OR "Bhutan*" OR "Bolivia*" OR "Bosnia*" OR "Botswan*" OR "Brazil*" OR "Burkina Faso" OR "Burundi*" OR "Cabo Verd*" OR "Cambodia*" OR "Cameroon*" OR "Central African Republic" OR "Chad" OR "China" OR "Colombia*" OR "Comoros" OR "Congo*" OR "Costa Rica*" OR "Côte d Ivoire" OR "Cuba*" OR "Republic of Korea" OR "Djibouti*" OR "Dominica*" OR "East Timor" OR "Ecuador*" OR "Egypt*" OR "El Salvador*" OR "Eritrea*" OR "Eswatini*" OR "Ethiopia*" OR "Fiji" OR "Gabon*" OR "Gambia*" OR "Gaza Strip" OR ("Georgia" NOT "state") OR "Ghan*" OR "Grenada" OR "Guatemal*" OR ("Guinea" NOT ("guinea pig*")) OR "Guyan*" OR "Haiti*" OR "Herzegovin*" OR "Hondur*" OR "India*" OR "Indones*" OR "Iran*" OR "Iraq*" OR "Ivory Coast" OR "Jamaica*" OR "Jordan*" OR "Kazakhstan*" OR "Kenya*" OR "Kiribat*" OR "Kosov*" OR "Kyrgyzstan*" OR "Lao*" OR "Leban*" OR "Lesoth*" OR "Liberia*" OR "Libya*" OR "Madagascar*" OR "Malaw*" OR "Malaysi*" OR "Maldives" OR "Mali" OR "Malian" OR "Marshall Islands" OR "Mauritani*" OR "Mauriti*" OR "Mexic*" OR "Micronesi*" OR "Mocambiqu*" OR "Moldov*" OR "Mongoli*" OR "Monteneg*" OR "Montserrat" OR "Morocc*" OR "Mozambiqu*" OR "Myanmar" OR "Namibi*" OR "Nauru" OR "Nepal*" OR "Nicaragu*" OR "Niger" OR "Nigeria*" OR "Niue" OR "North Korea*" OR "North Macedoni*" OR "Pakistan*" OR "Palau*" OR "Panam*" OR "Paragua*" OR "Peru*" OR "Philippin*" OR "Rhodesia*" OR "Rwand*" OR "Saint Helena" OR "Saint Lucia" OR "Saint Vincent" OR "St Helena" OR "St Lucia" OR "St Vincent" OR "Samoa*" OR "Sao Tome" OR "Senegal*" OR "Serbia*" OR "Sierra Leon*" OR "Solomon Islands" OR "Somali*" OR "South Africa*" OR "Southern Africa*" OR "Sri Lank*" OR "Sub Saharan Africa*" OR "Subsaharan Africa*" OR "Sudan*" OR "Surinam*" OR "Syria*" OR "Tajikistan" OR "Tanzani*" OR "Thai*" OR "Timor-Leste" OR "Togo*" OR "Tokelau" OR "Tonga" OR "Tunis*" OR "Turki*" OR "Turkmenistan" OR "Tuvalu" OR "Ugand*" OR "Ukrain*" OR "Uzbekistan" OR "Vanuatu" OR "Venezuel*" OR "Viet Nam*" OR "Vietnam*" OR "Wallis and Futuna" OR "West Bank" OR "Yemen*" OR "Zambia*" OR "Zimbabw*")	
#7	#6 NOT TI=("prenatal*" OR "pre-natal*" OR "fetal" OR "foetal" OR "fetus*" OR "foetus*")	818
#6	#4 AND #5	1,068
#5	TS=("resect*" OR "treat*" OR "surg*" OR "operat*" OR "invasive" OR "therap*" OR "chemo*" OR "manag*")	18,546,669
#4	(#1 AND #2) OR #3	1,679
#3	TS=(("sacrococcygeal" OR "coccygeal" OR "sacral") NEAR/3 "germ cell*")	49

Search	Query	Results
#2	TS=(("sacrococcygeal" OR "coccygeal" OR "sacral"))	29,351
#1	TS=("terato*")	52,663

Use reference:

Lobbestael, G. (2023). DedupEndNote (Version 1.0.1 20240114) [Computer software]. https://github.com/globbestael/DedupEndNote

Otten, R. De Vries, R. & Schoonmade, L. (2019). Amsterdam Efficient Deduplication (AED) method (Version 1). Zenodo. https://doi.org/10.5281/zenodo.3582928

Ouzzani, M., Hammady, H., Fedorowicz, Z., & Elmagarmid, A. (2016). Rayyan—a web and mobile app for systematic reviews. Systematic reviews, 5(1), 1-10. https://doi.org/10.1186/s13643-016-0384-4

Page MJ, McKenzie JE, Bossuyt PM, Boutron I, Hoffmann TC, Mulrow CD, et al. (2021) The PRISMA 2020 statement: An updated guideline for reporting systematic reviews. PLoS Med 18(3): e1003583. https://doi.org/10.1371/journal.pmed.1003583
