# Supplementary material for: Protocol for the development of a global core outcome set for the surgical treatment of sacrococcygeal teratoma in children: a systematic review and international Delphi study
Source: BMJ Open. 2026 Jan 16;16(1):e112492. doi: 10.1136/bmjopen-2025-112492 (PMC12815119; doi:10.1136/bmjopen-2025-112492)
Supplement: Supplementary file 3 [file bmjopen-2025-112492.reviewer_comments.pdf]

PEER REVIEW HISTORY

BMJ Open publishes all reviews undertaken for accepted manuscripts. Reviewers are asked to complete a checklist review form and are provided with free text boxes to elaborate on their assessment. These free text comments are reproduced below.

ARTICLE DETAILS

Title (Provisional)

Protocol for the development of a global core outcome set for the surgical treatment of sacrococcygeal teratoma in children: a systematic review and international Delphi study

Authors

Dongen, Malou Cécile; van Rijn, Rick; Sharma, Shilpa; Raphael, Martine F.; de Vries, Ralph; Abouzeid, Amr A.; Bugiani, Marianna; Chirdan, Lohfa B.; van Heurn, Ernest L.W.; Derikx, Joep P.M.; Kremer, Marijke E.B.; Steering Group, SCT-COS

VERSION 1 - REVIEW

**Reviewer**	**1**
**Name**	**Sekerci, Cagri**
**Affiliation**	**Marmara University**
**Date**	**13-Nov-2025**
**COI**	**None**

This manuscript addresses an important gap in the development of a core outcome set for the surgical management of sacrococcygeal teratoma. Also, the steps of the study protocol generation are clear and evidence-based.

However, several conceptual and methodological clarifications are needed prior to publication, such as;

1. The manuscript should describe how the draft of outcome items will be constructed from the systematic review findings (e.g. raw data collection, discussion parts of studies, future perspectives, questionnaires, combination?)

2. The decision is limited by the inclusion of pathologists and radiologists as stakeholder groups for clinical outcomes and long-term follow-up rather than clinical departments that care for those groups of patients, such as neurosurgeons, urologists, gastroenterologists, physiotherapists, etc.

3. The manuscript states that patients and caregivers will complete the survey after building the questionnaire, but as we know from the symptom score development studies, it is important to interview with them prior to the development of items to better reflect real-world experience.

4. Also, the outcome set may be categorised in sections to systematically assess and measure disease-related specific conditions such as gastrointestinal, genitourinary, neurological, musculoskeletal, and psychological.

5. The study protocol is well defined and clearly described, but a flow chart may be beneficial for readers in order to improve readability.

6. What is the exact sentence to be used and sent to experts during the Delphi process?

7. Please indicate the blinding method for experts for transparency.

8. A few spelling and grammar errors exist in the text, to be corrected before the revision.

VERSION 1 - AUTHOR RESPONSE

Reviewer: 1
Dr. Cagri Sekerci, Marmara University

Comment 1:
The manuscript should describe how the draft of outcome items will be constructed from the systematic review findings (e.g., raw data collection, discussion parts of studies, future perspectives, questionnaires, combination?)

Response 1:
We thank the reviewer for this important suggestion. In response, we have clarified the process for constructing the outcomes. The outcomes will be extracted from the included studies and recorded in a spreadsheet. Two independent reviewers (JD and MD) will then examine the outcomes, identify any overlapping terms, and categorize them based on their conceptual similarity. Only outcomes explicitly reported in the results sections of the included studies will be considered for inclusion. Outcomes mentioned in the discussion or future perspectives sections will not be included unless they are also presented as concrete outcomes in the results section. The grouped outcomes will be categorized into domains “Death”, “Pathophysiological Manifestations”, “Life Impact”, “Resource Use”, and “Adverse Events”. Additionally, the outcomes will be presented to the study’s Steering Group for review. Following their feedback, the refined outcome list will be used to create the Delphi questionnaire. This approach ensures that the final outcome list is both comprehensive and aligned with the Core Outcome Measures in Effectiveness Trials (COMET) methodology. We have updated the "Data Extraction" and "Data Synthesis" sections to reflect these steps.

Manuscript change:
Revisions made on page 3, lines 140–158

Comment 2:
The decision is limited by the inclusion of pathologists and radiologists as stakeholder groups for clinical outcomes and long-term follow-up rather than clinical departments that care for those groups of patients, such as neurosurgeons, urologists, gastroenterologists, physiotherapists, etc.

Response 2:
We thank the reviewer for this thoughtful comment. Radiologists and pathologists remain relevant as stakeholder groups given their role in assessing imaging and pathology at diagnosis and when recurrence is suspected during follow-up.
In line with the reviewer's comment, we recognise that several specialties contribute meaningfully to the long-term care of children with SCT. Urological sequelae following SCT surgery are consistently reported in the literature. We will therefore include paediatric urologists as their clinical perspective is essential when prioritising urological outcomes in the Delphi process. We acknowledge that gastrointestinal symptoms such as constipation or faecal incontinence are common in the long-term follow-up after SCT resection. In many centres including ours, these issues are largely managed by the general paediatrician. For this reason, we do not plan to recruit paediatric gastroenterologists as a separate group. Their expertise can still be represented through the open "other specialty" option in the survey, which allows participants to indicate additional disciplines when relevant. Neurosurgeons and physiotherapists may be involved in selected cases, but they are not routinely responsible for long-term follow-up in SCT. To keep the Delphi focused on the specialties most consistently involved, we will concentrate on paediatric surgeons, paediatricians, paediatric oncologists and paediatric urologists.

Manuscript change: Revisions made on pages 4-5, lines 188-200

Comment 3:
The manuscript states that patients and caregivers will complete the survey after building the questionnaire, but as we know from the symptom score development studies, it is important to interview with them prior to the development of items to better reflect real-world experience.

Response 3:
We appreciate the reviewer's comment and agree that involving patients and caregivers prior to the development of the questionnaire is essential to ensure that the items reflect real-world experiences. To address this, we plan to involve two groups of parents in testing the lay version of the questionnaire before the first Delphi round. Their feedback will be used to refine the questionnaire, ensuring that the outcomes we measure are both relevant and understandable. Additionally, during this testing phase, parents will have the opportunity to suggest any additional outcomes that were not identified in the systematic review. If important outcomes are identified through this process, they will be incorporated into the final version of the questionnaire. This ensures that the questionnaire is directly shaped by the perspectives of patients and caregivers.

Manuscript change: Revisions made on page 5, lines 223-227

Comment 4:
Also, the outcome set may be categorised in sections to systematically assess and measure disease-related specific conditions such as gastrointestinal, genitourinary, neurological, musculoskeletal, and psychological.

Response 4:
We thank the reviewer for this suggestion. We agree that categorizing the outcome set into sections such as gastrointestinal, genitourinary, neurological, musculoskeletal, and psychological conditions could be helpful. However, we would like to clarify that the OMERACT domains already comprehensively cover these categories. As seen in previous Core Outcome Set (COS) studies (e.g., Hall et al., 2017; Knaapen et al., 2020), OMERACT remains the primary framework for classifying outcomes. Given the greater heterogeneity of outcomes in SCT, we do see value in presenting the outcome list in subcategories, such as urological, gastrointestinal, and neurological outcomes. After careful consideration, we have concluded that further categorization might be too detailed for this protocol and will not be implemented at this stage.

Comment 5:
The study protocol is well defined and clearly described, but a flow chart may be beneficial for readers in order to improve readability.

Response 5:
A flowchart will indeed enhance the readability of the protocol and provide a clearer overview of the study design. We added a flowchart and referred to in the methods section as Figure 1.

Comment 6:
What is the exact sentence to be used and sent to experts during the Delphi process?

Response 6:
The Delphi survey will ask all stakeholders the following question: *"How important is this outcome for inclusion in a Core Outcome Set?"* Each outcome will be rated on a 9-point scale: 1 to 3 (not important), 4 to 6 (important, but not essential), and 7 to 9 (essential).

Manuscript change: Revisions made on page 6, lines 242-243

Comment 7:
Please indicate the blinding method for experts for transparency.

Response 7:
The Delphi survey will be conducted anonymously. Participants do not see each other's individual ratings at any point. After each round, they receive only a summary of the group results, including the median score and the distribution of ratings for each outcome. This provides feedback without revealing individual responses, which allows each participant to reassess the outcomes independently in the next round.

Manuscript change: Revisions made on page 6, lines 232-234

Comment 8:
A few spelling and grammar errors exist in the text, to be corrected before the revision

Response 8:

We have corrected the spelling and grammar errors in the manuscript, and we have ensured consistency throughout the text.
